# A Novel Lipid Extraction Method from Wet Microalga *Picochlorum* sp. at Room Temperature

**DOI:** 10.3390/md12031258

**Published:** 2014-03-06

**Authors:** Fangfang Yang, Wenzhou Xiang, Xiumei Sun, Hualian Wu, Tao Li, Lijuan Long

**Affiliations:** 1 Key Laboratory of Tropical Marine Bio-resources and Ecology, South China Sea Institute of Oceanology, Chinese Academy of Sciences, Guangzhou 510301, China; E-Mails: ycuyang@163.com (F.Y.); xwz@scsio.ac.cn (W.X.); sunxm@scsio.ac.cn (X.S.); hlwu@scsio.ac.cn (H.W.); taoli@scsio.ac.cn (T.L.); 2 Graduate School of Chinese Acedemy of Sciences, Beijing 100049, China

**Keywords:** lipid extraction, wet microalga, biofuel, ethanol, *Picochlorum* sp.

## Abstract

A novel method using ethanol was proposed for extracting lipids from wet microalga *Picochlorum* sp. at room temperature and pressure. In this study, Central Composite design (CCD) was applied to investigate the optimum conditions of lipid extraction. The results revealed that the solvent to biomass ratio had the largest effect on lipid extraction efficiency, followed by extraction time and temperature. A high lipid extraction yield (33.04% of the dry weight) was obtained under the following extraction conditions: 5 mL solvents per gram of wet biomass for 37 min with gentle stirring at room temperature. The extraction yield was comparable to that obtained by the widely used Bligh-Dyer method. Furthermore, no significant differences in the distribution of lipid classes and fatty acid composition were observed according to different extraction methods. In conclusion, these results indicated that the proposed procedure using ethanol could extract lipids from wet biomass efficiently and had giant potential for lipid extraction at large scale.

## 1. Introduction

Due to the serious energy crisis and environmental pollution associated with the using of fossil fuels, biofuel derived from microalgae has been advocated in recent years. Compared to other feedstocks like plant oils, animal fats, *etc.*, microalgae have outstanding advantages: they are capable of growing rapidly and converting CO_2_ into substantial amounts of lipids [1,2,3]. Some microalgae species can absorb essential nutrients including the carbon, nitrogen and phosphorus from exhaust gas and waste water [4,5]. Additionally, many microalgae can grow well in unfavorable lands and saline water. That is, microalgae do not compete for the land required for producing food and overcome the discord between food and fuel [6,7,8].

Extracting lipids is one of the most key and limited processes for biofuel production based on microalgae at large scale. The conventional methods for lipid extraction generally involve dewatering before extracting lipids since residual water in wet microalgal biomass hindered mass transfer of the lipids from the cell and then lead to a decrease in the efficiency of lipids extraction. Lardon *et al.* [9] and Patil *et al.* [10] reported that the consumption energy of the drying accounted for the majority of the total process energy (84.9%). In addition, the organic solvents used in the conventional methods are regarded as highly-toxic, being environmentally unfriendly. These shortcomings hinder the application of conventional methods in industrial lipid extraction, despite of the high extraction efficiency. Therefore, it is essential to develop novel approach of lipid extraction, which is an effective eco-friendly process. 

Compared to the traditional methods involving drying, extracting lipids from wet biomass is a more economic method, which requires no energy to dry the biomass. Various researchers have investigated the wet lipid extraction methods, including ultrasound-assisted extraction [11], simultaneous distillation and extraction process [12], microwave-assisted extraction [13] and supercritical fluid extraction [14]. Unfortunately, the most cases still require high temperature, long times or high energy inputs. Therefore, the technologies of lipid extraction are only limited to laboratory scale. The ideal method being suitable for industrial-scale extraction has not yet been settled. 

The aim of the present study is to develop an eco-friendly solvent technique that will make it possible to extract lipid from wet microalgal biomass efficiently at large scale. Ethanol is considered as a cheap and safe solvent; additionally, ethanol has a strong affinity to the lipid complex, which implies that lipids can be extracted efficiently. Fajardo *et al.* [15] used ethanol, following by hexane, to extract and purify lipids from drying microalga *Phaeodactylum tricornutum* efficiently. Chen *et al.* [16] extracted lipids from wet microalga *Nannochloropsis* sp. at high temperature and pressure. However, no investigation about the application of ethanol in extracting lipids from wet microalgal biomass at room temperature and pressure has been reported.

In this study, we adopted a novel method using ethanol with gentle stirring for lipid extraction from wet microalga *Picochlorum* sp. directly. Then the effects of parameters including time, temperature, and the ratio of solvent to biomass on the lipid extraction yield were investigated by Central Composite design (CCD) to identify the optimum extraction conditions. Finally, the proposed methods were compared with the conventional Bligh-Dyer method in terms of lipid extraction yield, lipids quality.

## 2. Results and Discussion

### 2.1. Examine Ethanol for Lipid Extraction from Wet Biomass

A novel method, extracting lipids from wet biomass using ethanol at room temperature (27 °C) for 30 min, was proposed. The ratio of ethanol to wet biomass was 4:1 (mL/g). To examine the feasibility of the method, the lipid extraction yield was determined as described in Section 3.3. The lipids arising from microalgal biomass by ethanol, referred to as crude lipids, frequently contain several non-lipids (proteins bonding to lipids strongly and carbohydrates). To avoid the interference of non-lipid complex, the crude lipids were purified. Hexane was a low-toxicity solvent and employed to remove non-lipids complex from crude lipids. The purified lipids were quantified and used to calculate the lipid extraction yield. The extraction yield by ethanol was close to that of Bligh-Dyer’s method, namely, 31.89% and 33.18% of the dry weight, respectively. That is, the extraction rate of lipids was 96.1%. The result implied that ethanol had potential for extracting lipids from wet microalgae at room temperature. 

### 2.2. Investigating the Optimum Procedure of Extracting Lipids Using Ethanol

In the experiment, Central Composite design (CCD) was employed to analyze comprehensively the influences of three extraction parameters on the lipid extraction yield and determine the optimum extraction conditions. Table 1 shown the actual factor levels corresponding to the coded factor levels. In total, 20 experiments were designated. 

**Table 1:** Levels and variables involved in Central Composite design.

Variables	Levels				
	−α	−1	0	1	α
Extraction time (min)	2.5	10	25	40	47.5
Extraction temperature (°C)	20	25	35	45	50
the ratio of solvent to biomass (mL/g)	1	2	4	6	7

The corresponding response value obtained from each run were illustrated in Table 2. By analyzing these data in Table 2, the following second order polynomial equation expressed in terms of coded values fitted to the results from the optimization experiments was obtained.

*Y* = +30.98 + 1.27 *X*_1_ + 0.042*X*_2_ + 4.06*X*_3_ + 0.14*X*_1_*X*_2_ − 0.24*X*_1_*X*_3_ − 0.25*X*_2_*X*_3_ − 0.66*X*_1_^2^ + 0.37*X*_2_^2^ − 2.71*X*_3_^2^

where, *Y* stood for lipid extraction yield (% of the dry weight); X_1_, *X*_2_ and *X*_3_ were extracting time (min), extracting temperature (°C) and the ratio of solvent to biomass (mL/g), respectively.

To check the adequacy of the quadratic polynomial model, the statistical significance of the above equation was calculated, illustrated in Table 3. Here, *R*^2^ was 0.9857, indicating that 98.57% of the data in CCD could be explained by the response surface model, that is, the model can be carried out to reveal the effects of variables on the response value and predict the maximum response value in subsequent optimization experiments. In addition, the *F*-value of 76.57 demonstrated that the model was significant, as indicated by the *p*-value less than 0.0001, which further supported the fitness of the proposed model. From the analysis of *R*_adj_^2^ and *R*_pred_^2^, there was a high degree of agreement between them. In conclusion, these results clearly indicated that the model could be used to explain these data well.

**Table 2:** Results and experimental layout in Central Composite design.

NO.	Extraction time (min)	Extraction temperature (°C)	Solvent to biomass ratio (mL/g)	Extraction yield (of the dry weight)	
				Experimental	Predicted
1	10.00	25.00	2.00	22.62	22.26
2	40.00	25.00	2.00	25.20	25.01
3	10.00	45.00	2.00	22.77	22.57
4	40.00	45.00	2.00	26.26	25.87
5	10.00	25.00	6.00	30.84	31.36
6	40.00	25.00	6.00	32.81	33.14
7	10.00	45.00	6.00	30.35	30.67
8	40.00	45.00	6.00	32.51	33.00
9	2.50	35.00	4.00	27.73	27.60
10	47.50	35.00	4.00	31.51	31.41
11	25.00	20.00	4.00	31.90	31.76
12	25.00	50.00	4.00	31.96	31.88
13	25.00	35.00	1.00	17.97	18.79
14	25.00	35.00	7.00	32.01	30.96
15	25.00	35.00	4.00	31.29	30.98
16	25.00	35.00	4.00	30.11	30.98
17	25.00	35.00	4.00	30.56	30.98
18	25.00	35.00	4.00	31.28	30.98
19	25.00	35.00	4.00	31.64	30.98
20	25.00	35.00	4.00	30.83	30.98

**Table 3:** Statistical analysis for experimental results of Central Composite design.

Source	Sum of squares	Df	Mean square	*F* value	*p*-Value
Model	308.98	9	34.33	76.57	< 0.0001
Linear					
X_1_	20.16	1	20.16	44.97	< 0.0001
X_2_	0.022	1	0.022	0.049	0.8301
X_3_	205.72	1	205.72	458.83	< 0.0001
Quadratic					
X_1_^2^	0.15	1	0.15	0.34	0.0105
X_2_^2^	0.47	1	0.47	1.05	0.1041
X_3_^2^	0.50	1	0.50	1.12	< 0.0001
Interaction					
X_1_X_2_	4.43	1	4.43	9.87	0.5714
X_1_X_3_	1.43	1	1.43	3.20	0.3301
X_2_X_3_	75.84	1	75.84	169.16	0.3148
Residual	4.48	10	0.45		
Lack of fit	2.91	5	0.58	1.85	0.2578
Pure error	1.57	5	0.31		
Cor total	313.47	19			

*R*^2^ = 0.9857; *R*_Adj_^2^ = 0.9728; RPred2 = 0.9223.

As shown in Table 3, the linear coefficient indicated that the ratio of solvent to biomass (X_3_) was the most significant independent variable impacting on extraction yield with *p*-value less than 0.01. The higher ratio of solvent to biomass was, the more the extraction yield could be. The extraction time (X_1_) also exerted a positive individual influence on the extraction yield. That implied that an increase in the extraction time improved the lipid extraction amounts. In addition, the ratio of solvent to biomass and extraction time exerted the significant quadratic effects (Table 3). However, other terms (*X*_2_, *X*_2_^2^, *X*_1_*X*_2_, *X*_1_*X*_3_, *X*_2_*X*_3_) were insignificant (Table 3). In particular, extraction temperature in the range of 20–50 °C had litter effect on extraction yield and the lipids could be extracted effectively at room temperature. It was therefore possible that *Picochlorum* sp. was cracked during the extracting process and ethanol get into the cell easily without the cell wall resistance. Additionally, gentle stirring could accelerate cells lysis and elevate extraction efficiencies. 

**Figure 1:**
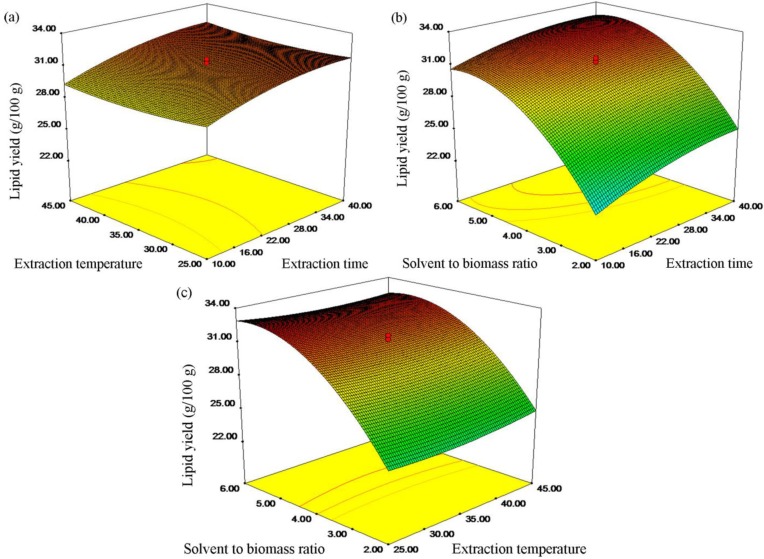
Response surfaces and contour plots showing the mutual effect of (**a**) extraction temperature and time; (**b**) the ratio of solvent to biomass and extraction time; (**c**) the ratio of solvent to biomass and extraction temperature on the lipid extraction yield.

To understand the interaction of the corresponding parameters, the regression model was represented in terms of response surface plots, as shown in Figure 1. Figure 1a represented the mutual effect of the extraction time and temperature on the extraction yield. It was apparent that the interaction between the two selected variables had litter influence on the extraction yield. As seen in Figure 1b, it could be observed that the extraction yield increased significantly with an increase in the ratio of solvent to biomass at a given extraction time. However, excess solvent amount would not improve further the extraction yield. In addition, as extraction time elevating, the extraction efficiency enhanced, resulting in a higher lipid extraction yield. However, the interaction terms of the two variables possessed litter role as indicated by its *p*-value of 0.3301. Similarly, the interaction between extraction temperature and the ratio of solvent to biomass had insignificant effects on extraction yield, as illustrated in Figure 1c.

### 2.3. The Validation of the Model

According to the above results, the optimum extraction conditions were obtained as following: the ratio of solvent to biomass was 5:1 (mL/g) at room temperature (26 °C) for 37 min of extraction time. The extraction yield obtained from *Picochlorum* sp. was predicted to be 33.10% of the dry weight. In order to confirm these conclusions, extraction experiments based on the optimal extraction parameters were performed and the extraction yield was determined. The experimental value was 33.04% of the dry weight, which was agreement well with the predicted value calculated by the model equation, demonstrating the adequacy of the regression equation. The results also revealed that ethanol could employed successfully to extract lipids from wet microalga *Picochlorum* sp. However, the particular reason for this is not well understood and requires further research. Halim *et al*. [17] proposed a probable mechanism for lipid extraction from microalgae by solvent. The solvent penetrated through the cell membrane into the cytoplasm. Then the solvent interacted with the lipid complex and formed a complex. Finally, the solvent-lipids complex diffused out the cell and lipids were extracted. Ranjan *et al*. [18] revealed that the prominent mechanism of lipid extraction by organic solvent was diffusion across a cell wall. The extent of diffusion was attributed to the selectivity of the solvent. In the study, ethanol was used as extractant. Since ethanol had both polar and non-polar properties, it could interact with non-poplar and poplar lipids after entering into cells. This meant that ethanol could pull out neutral and polar lipids from cell efficiently [15,19]. Additionally, the gentler stirring in the extraction process could sweep away the extracted lipids from the microalgal cell surface and maintain a continuous diffusion of lipids from the cells. On the other hand, the disruption of microalgal cells was also a mechanism of lipid extraction [18]. The disruption depended greatly on cell morphology and the probability of interaction of the cell with cavitation bubbles [18,20]. The cell wall of *Picochlorum* sp. used in this study could be disrupted in the extracting procedure and cellular contents were probably released. Therefore, ethanol could extract lipids from wet microalga efficiently. However, the special mechanism requires further research.

### 2.4. Lipid Analysis and Fatty Acid Composition Comparisons

To evaluate the efficiency of our proposed method, extracting lipids from wet biomass using ethanol, the conventional Bligh-Dyer method was employed as a reference, due to its high lipid extraction efficiency. The moisture content of wet biomass was 90.02% wet weight. 

Table 4 presented the lipid extraction yield for two extraction methods. When using ethanol as extractant, a high extraction yield of lipids (33.04% of the dry weight), which was similar with that of Bligh-Dyer method (33.18% of the dry weight), was obtained. This implied extraction rate of the proposed method was up to 99.6%. Additionally, we investigated the lipid extraction ratio of the fresh microalga. The results revealed that there were no significant changes in the extraction efficiency.

**Table 4:** Fatty acid profile comparison between different extraction methods.

	Extraction method	
	Bligh-Dyer	Ethanol
Lipid extraction yield (% of the dry weight)	33.18 ± 0.24	33.04 ± 0.16
Fatty acid composition (% of FAME)		
Saturated		
C16:0	32.49 ± 1.54	29.48 ± 3.12
C18:0	2.82 ± 0.43	6.00 ± 1.89
Unsaturated		
C16:1	2.57 ± 0.62	2.05 ± 0.38
C16:2	5.76 ± 0.16	5.65 ± 0.58
C16:3	6.62 ± 0.54	6.09 ± 0.11
C18:1	8.42 ± 0.01	9.06 ± 0.28
C18:2	22.25 ± 0.21	22.47 ± 2.63
C18:3	17.37 ± 0.65	17.04 ± 0.93
Others	1.71 ± 0.35	2.16 ± 0.40

The major fatty acid composition of lipids was determined by gas chromatography coupled to mass spectrometry (GC-MS). As shown in Table 4, no significant differences in fatty acid composition were observed between different extraction methods. The dominant fatty acids were palmitic acid (C16:0), oleic acid (C18:1), linoleic acid (C18:2) and linolenic acid (C18:3), which accounted for approximately 80% of total fatty acids. Other fatty acids, such as palmitolenic (C16:1), palmitolenic (C16:2), palmitoleidic (C16:3) and stearic (C18:0), were also present in smaller quantities. The fatty acid composition of *Picochlorum* sp. was similar with the profile presented by Tanzi *et al.* [12]. In generally, C16:0, C18:0, C18:1 and C18:2 were known to the most common components in biodiesel [14,21]. Therefore, the extracted lipids of *Picochlorum* sp. were suitable for biodiesel production.

The content of each fatty acid had no significant difference between two methods according to statistical analysis (*P* > 0.05). However, the ratio of some fatty profiles, especially C18:0, represented slight variation according to different extraction methods. When using ethanol as solvent, the lower proportion of C16:0, C16:1 and C16:3, with corresponding increasing in C18:0 and C18:1, compared to the Bligh-Dyer method (Table 4). The probable reason was that the change of extraction conditions resulted in the fatty acid profile of the extracted lipids [14]. The results revealed that ethanol could extract most of fatty acids efficiently. In addition, it must be highlighted that the microalga *Picochlorum* sp. had a high percentage of linolenic acid (around 17%), which was an essential and important fatty acid to human health [22]. 

The lipid class was determined and presented as % of lipid class in total lipids as shown in Table 5. The lipids were consisted mainly of neutral lipid, glycolipid and phospholipid. By statistical analysis, no significant difference in the content of each lipid class was observed (P > 0.05), although the content of phospholipid was higher than that obtained using Bligh-Dyer method. Additionally, the percentage of neutral lipid was highest (about 50% of total lipids) using the two extraction methods, suggesting the microalga *Picochlorum* sp. was a promising feedstock for biodiesel production. As a conclusion, most of essential lipids for biodiesel production could be extracted from wet biomass effectively using ethanol.

**Table 5:** The lipid class comparison between different extraction methods. Values were given as means of total lipids percentage ± standard deviation.

Lipid class	Extraction method	
	Bligh-Dyer	Ethanol
Neutral lipid	54.73 ± 1.47	53.49 ± 2.11
Glycolipid	16.46 ± 0.76	15.62 ± 0.54
Phospholipid	28.81 ± 0.71	30.89 ± 1.57

### 2.5. Lipid Extraction at Larger Scale and Ethanol Recycling

**Figure 2:**
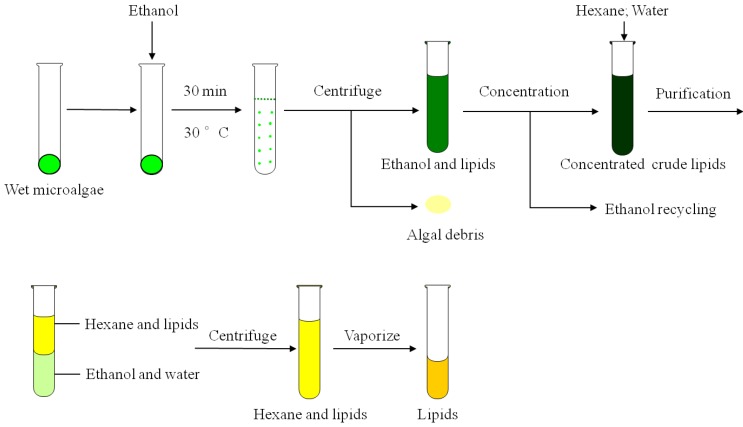
A scheme illustration of lipid extraction procedure from wet microalga using ethanol.

To validate the applicability of the optimal method in lipid extraction at enlarged scale, 250 g wet biomass was employed to extract lipids. A summary of the protocol was shown in Figure 2. A high extraction ratio of 99.4% was obtained, implying that the optimum method was effective for extracting lipids at larger scale. Additionally, ethanol was recovered by using distillation tower in order to decrease the consumption of ethanol. The results revealed that the recovery of ethanol reached a yield of 95.24% with the purity of 93%. Furthermore, the experiments confirmed that the recycled ethanol had high efficiency for extracting lipids from wet biomass. Therefore, the extraction method with ethanol was suitable for extracting lipids from microalgae at large scale, with a high extraction efficiency and low environment pollution. 

### 2.6. Lipid Extraction Methods Comparison

The lipid extraction method using ethanol was evaluated in terms of extraction yield and lipid quality. There was no significant difference compared to the conventional Bligh-Dyer method, that is, ethanol could extract lipids from wet microalga effectively. 

Comparing to the Bligh-Dyer method, the method, because it used ethanol, was environmentally friendly. Moreover, since the debris contained high contents of proteins, the debris would be reused for producing bait after extracting lipids. Conversely, the debris would be toxic and be not reused if the Bligh-Dyer method was employed to extract lipids. Therefore, the method was considered safe and suitable for the lipid extraction in the industrial scale. 

So far, there have been a few reports related to the application of ethanol in extracting lipids [15,16]. However, in these cases, dewatering or high temperature still be required. In the study, ethanol was employed successfully to extract lipids from high-moisture microalgae at room temperature and pressure. It was probable that the *Picochlorum* sp. used in this study had different bio-characteristics and the cell wall was cracked easily. Hence, there was slight cell wall resistance and the ethanol could enter into cells for extracting lipids quickly. This was consistent with Prommuak *et al.* [20] which revealed that the extraction efficiency depended greatly on cell morphology. Compared to the conventional Bligh-Dyer method, the method did not require dewatering and heating, which implied the method was easier to operate at large scale. 

In conclusion, the method based on ethanol possessed important advantages over Bligh-Dyer method, such as shorter treatment time, less environment pollution, high extraction efficiency, which was more applicable for lipid extraction at large scale than the conventional methods. 

Despite this, the effectiveness of the method will be further evaluated using different types of microalgae at large scale to test the practical application of this method. Otherwise, it would be necessary to further investigate the applications of the residual biomass and linolenic acid as byproduct, to improve the overall economics of microalgal biofuel production.

## 3. Materials and Methods

### 3.1. Strain, Culture Conditions and Harvesting Method

*Picochlorum* sp., isolated from the India Ocean, was cultured in the modified f/2 media composed of sea water with an addition of phosphorus and nitrogen sources. The cultures were cultivated in an outdoor raceway system of up to 500 m^2^. Temperature and illumination intensity depended on the daily weather. Daily microscopic analysis revealed that *Picochlorum* sp. was not contaminated. At the same time, microalgal cultures were harvested and concentrated by the floatation method. A dilute aqueous suspension with water content of 90.02% wet weight was obtained and stored at 4 °C for subsequent analysis. In the experiments involving dried microalgae, the concentrated microalgal cultures were frozen-dried completely in a lyophilizer. In the experiments where wet microalgae were used, the concentrated microalgal cultures were used to extract lipid directly without further treatment.

### 3.2. Conventional Lipid Extraction Method

The lipids of drying biomass were extracted by Bligh-Dyer’s method with chloroform and methanol mixture [23]. The method was used as standard to evaluate our proposed method of extracting lipids from wet biomass. The chloroform and methanol mixture was added to 100 mg of drying biomass at 50 °C for 1 h. The extracted lipids were quantified and analyzed.

### 3.3. Lipid Extraction using Ethanol as Extractant

Ethanol was performed to extract lipids from wet biomass (approximately 1 g). The lipids arising from microalgal biomass by ethanol, referred to as total lipids or crude lipids, frequently contain several non-lipids (proteins bonding to lipids strongly and carbohydrates). Then the biomass residue was removed and the crude lipids obtained were later purified. 

The lipid extraction ratio obtained by hexane was low since hexane preferably extracted non-polar lipids in the microalga. However, hexane was a low-toxicity solvent and could remove non-lipids from crude lipids efficiently [20]. In the study, to purify the crude lipids, the water and hexane were added into the crude lipids to form a liquid-liquid separation state according to the method illustrated by Fajardo *et al*. [15]. The upper phase (hexane and some ethanol) was loaded with most of the lipids while the lower phase (most ethanol with water) contained most non-lipids. The upper phase that contained lipids was transferred to a weighted tube and dried by stream of N_2_. The purified lipids were quantified and were later analyzed by gas chromatography and silica gel column chromatography. In all experiments, three parallels were set up for each treatment.

### 3.4. Experimental Design and Data Analysis

In order to investigate the optimum extraction conditions, a mathematical model-Central Composite design (CCD) was utilized when ethanol was used as extractant. By applying the CCD, the effect of each parameter, such as extraction time, extraction temperature and the ratio of solvent to biomass, on extraction yield were evaluated quickly and effectively. Table 1 illustrated the actual factor levels corresponding to the coded factor levels. In the experiment, the low and high levels of all variables, that is, star points were set up first. Depending on the number of parameters involved and desire of the design, the value of α, which expressed distance between star points and center points, was determined. In all, twenty experiments were employed with fifteen being the different combinations of three parameters and five being replications of center points. Each trial was performed in triplicate and lipid extraction yield was the mean values. According to the experimental results obtained, the second-degree polynomial equation was given below, which could calculate the predicted value of lipid extraction yield.

*Y* = α_0_ + α_1_*X*_1_ + α_2_*X*_2_ + α_3_*X*_3_ + α_4_*X*_1_*X*_2_ + α_5_*X*_1_*X*_3_ + α_6_*X*_2_*X*_3_ + α_7_*X*_1_^2^+ α_8_*X*_2_^2^+ α_9_*X*_3_^2^

where, *Y* represents the predicted value of extraction yield (% of the dry weight); *X*_1_, *X*_2_, *X*_3_ are the code values of extraction time (min), extraction temperature (°C) and the ratio of solvent to wet biomass (mL/g), respectively; α_0 _is quantity; α_1_–α_9_ stand for coefficient estimate.

The software (Design Expert, version 8.05) was conducted to analyze and calculate these results. Under the optimum conditions, the lipids were extracted. By comparing the experimental and predicted values, the model was verified.

### 3.5. Esterification and Analysis of Fatty Acids

The lipids were converted to fatty acid methyl ester (FAME) for gas chromatography. 1 mL of chloroform containing 0.2 mg of heptadecanoic acid (C17:0) was added to each of the purified lipid samples as an internal standard. Then 1 mL of NaOH-CH_3_OH was added at 75 °C for 10 min and 2 mL of BF_3_-CH_3_OH was added for transesterification reaction at 75 °C for 10 min. After the reaction, 3 mL of hexane and 1 mL of deionized water were added to the above samples. Finally, the samples were centrifuged and upper layer was separated for GC-MS analysis. Fatty acid compositions of the lipids were analyzed by GC-MS with an Omegawax 250 polyethylene glycol capillary column (length 30 m, diameter 0.25 mm and 0.25 μm film thickness) using the method reported by Goldberg *et al.* [24]. Samples of 1 µL were injected into the capillary column with a split ratio of 5:1. Helium was employed as the carrier gas with a flow rate of 1.5 mL/min. The temperatures of injector and detector both maintained at 250 °C. The column temperature was programmed from 130 °C at 5 °C/min ramp rate to 250 °C maintained for 5 min. Each component was identified by comparing retention time and fragmentation with standards using the GC-MS library. The fatty acid content was expressed as percentage of total fatty acids.

### 3.6. Lipid Analysis Using Silica Gel Column Chromatography

Lipid class separation was performed by silica gel column chromatography according to the method illustrated by Christie [25]. Typically, the samples of lipids re-suspended in chloroform were loaded onto silica gel column chromatography (Agela, Tianjin, China). Neutral lipid, phospholipid and glycolipid were successively eluted using chloroform, acetone and methanol, respectively. Each component was dried by stream of N_2_ and then weighed. 

### 3.7. Lipid Extraction at Larger Scale and Ethanol Recycling

Ethanol was added to wet microalgal biomass (250 g) in an approximately 5:1 mass ratio. Under the optimum conditions, lipids were extracted. After extraction, the supernatant, loading with lipids and ethanol, was separated and then ethanol was recycled using distillation tower. The purity of ethanol was measured by the alcohol detector. The recycled ethanol was once again employed to extract lipids from wet biomass in order to evaluate its ability of extracting lipids. A summary of the protocol was shown in Figure 2. 

## 4. Conclusions

This study demonstrated that the novel method using ethanol could be used to extract lipids from high-moisture microalgae at room temperature, with an extraction yield of 33.04% of the dry weight. The extraction yield was comparable to that of the conventional Bligh-Dyer method. Additionally, only minor variations in lipid profiles and fatty acid composition were observed according to different methods, suggesting that ethanol extracted the main components for biodiesel production effectively. Further research revealed that a high lipid extraction ratio of 99.4% was obtained using the proposed method at larger scale. Taken together, the results suggested that the method with ethanol was an easy, less environmentally polluting and high efficiency extraction process.
